# Viability of *Legionella pneumophila* in Water Samples: A Comparison of Propidium Monoazide (PMA) Treatment on Membrane Filters and in Liquid

**DOI:** 10.3390/ijerph14050467

**Published:** 2017-04-27

**Authors:** Sara Bonetta, Cristina Pignata, Silvia Bonetta, Lorenza Meucci, Donatella Giacosa, Elena Marino, Giorgio Gilli, Elisabetta Carraro

**Affiliations:** 1 Department of Public Health and Pediatrics, University of Torino, P.zza Polonia 94, 10126 Torino, Italy; cristina.pignata@unito.it (C.P.); silvia.bonetta@unito.it (S.B.); giorgio.gilli@unito.it (G.G.); elisabetta.carraro@unito.it (E.C.); 2 Società Metropolitana Acque Torino S.p.A., C.so XI Febbraio, 14, 10152 Torino, Italy; lorenza.meucci@smatorino.it (L.M.); donatella.giacosa@smatorino.it (D.G.); elena.marino@smatorino.it (E.M.)

**Keywords:** *Legionella*, water, propidium monoazide (PMA), quantitative PCR (qPCR)

## Abstract

*Legionella pneumophila* is a ubiquitous microorganism widely distributed in aquatic environments and can cause Legionellosis in humans. A promising approach to detect viable cells in water samples involves the use of quantitative polymerase chain reaction (qPCR) in combination with photoactivatable DNA intercalator propidium monoazide (PMA). However, the PMA efficiency could be different depending on the experimental conditions used. The aim of this study was to compare two PMA exposure protocols: (A) directly on the membrane filter or (B) in liquid after filter washing. The overall PMA-induced qPCR means reductions in heat-killed *L. pneumophila* cells were 2.42 and 1.91 log units for exposure protocols A and B, respectively. A comparison between the results obtained reveals that filter exposure allows a higher PMA-qPCR signal reduction to be reached, mainly at low concentrations (*p* < 0.05). This confirms the potential use of this method to quantify *L. pneumophila* in water with low contamination.

## 1. Introduction

*Legionella* is a ubiquitous microorganism that is widely distributed in aquatic environments. From natural reservoirs, it can reach and colonize artificial aquatic environments such as the water system of buildings, cooling towers, evaporative condensers, and dental unit waterlines [1,2,3,4]. *Legionella pneumophila* is the species most frequently found in human disease, causing Legionnaires’ disease or the flu-like Pontiac fever in humans, through inhalation of contaminated water aerosols [5].

Environmental monitoring represents an important tool for the assessment of *Legionella* contamination. Assessments of *L. pneumophila* in water are typically performed by culture isolation on selective media. However, although *Legionella* culture growth is essential for identifying and typing *Legionella* strains, it has several limits including the long incubation times required for the growth of colonies, the simultaneous growth of other bacteria, and the inability to detect the viable but non-culturable bacteria (VBNC) that may represent a public health hazard [6]. Quantitative polymerase chain reaction (qPCR) has been proposed as an alternative tool for rapid, sensitive, and specific detection of *Legionella* in water samples. This method may overcome many disadvantages of traditional culture methods; however, qPCR does not allow viable cells to be distinguished from dead cells [7]. A promising approach for detecting viable cells involves the use of qPCR along with photoactivatable DNA intercalators, either ethidium monoazide (EMA) or propidium monoazide (PMA), which can penetrate the membrane of compromised cells and block PCR amplification [8,9]. Notably, PMA has shown a strong specificity for dead cells compared to EMA [10]. Only a few studies have used a PMA-qPCR method to detect *Legionella* in water samples, but comparisons of these results are difficult because different concentrations, light exposure times, and PMA exposure protocols were employed [2,6,11,12]. Moreover, different studies showed that the PMA efficiency could be different in each experimental condition and therefore needs to be tested in order to guarantee the reliability of results [11].

The purpose of our study was to investigate whether the exposure to PMA in a different step of the method could affect the results obtained with this photoactivatable dye. To this end, the results obtained exposing *Legionella* cells to PMA directly on the membrane filter (Exposure Protocol A) were compared with PMA exposure in liquid after filter washing (Exposure Protocol B).

## 2. Materials and Methods

### 2.1. Bacterial Strain and Cultivation

*L. pneumophila* (ATCC 33152) was grown at 37 °C on BYEα broth.

To obtain a bacterial suspension of *L. pneumophila* in log phase, 100 µL of *Legionella* broth culture was inoculated into BYEα (150 mL) and incubated with shaking at 200 rpm until the culture reached an OD 650 of ~0.1 (corresponding to ~ 1 × 10^7^ colony-forming units (CFU)/mL considering the growth curve previously evaluated). The cell concentration was confirmed by plate counting in triplicate on BCYEα agar. At the same time, an aliquot of the bacterial suspension (~1 × 10^7^ CFU/mL) was incubated at 90 °C for 20 min in order to obtain heat-killed bacteria (0% cultivable). The complete loss of culturability after the heat treatment was verified by spreading 100 µL onto BCYEα agar plates followed by incubation at 37 °C.

### 2.2. Cytotoxic Effect of PMA

PMA (Biotium, Hayward, CA, USA) was dissolved in 20% dimethylsulfoxide (DMSO) to obtain a stock concentration (20 mM) and stored at −20 °C in the dark.

In order to evaluate the cytotoxic effect of PMA, preliminary tests were carried out using different concentrations of PMA. Aliquots of *L. pneumophila* (~10^6^ CFU/mL in the log phase) were treated with 25, 12.5, or 7 µM of PMA directly applied to the filter or in saline solution. Each concentration of PMA was tested three times with both methods. Cells without PMA treatment were used as a control. The filter was sonicated (7 min) in 10 mL of saline solution. Effects on bacteria were verified by culture in triplicate on BCYEα agar (37 °C). The detection limit of the culture method using the membrane filtration was 100 CFU/L.

### 2.3. Exposure Protocols and Quantification by qPCR and PMA-qPCR

**Figure 1:**
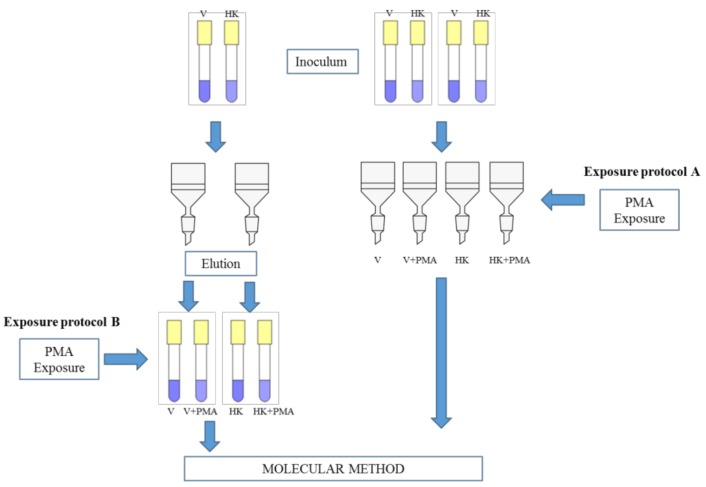
Scheme of the exposure protocols used. V: viable *Leginonella pneumophila*; HK: heat-killed *Leginonella pneumophila*; PMA: propidium monoazide.

The two exposure protocols are reported in Figure 1. Briefly, in Exposure Protocol A, *Legionella* cells were exposed to PMA directly on the membrane filter; in Exposure Protocol B, *Legionella* cells were exposed to PMA in liquid after filter washing.

For both methods, a bacterial suspension (viable or heat-killed *L. pneumophila* cells) in the log phase, prepared as reported above, was serially diluted to obtain suspensions that contained 1 × 10^6^, 1 × 10^5^, 1 × 10^4^, 1 × 10^3^, and 1 × 10^2^ CFU/mL. Each suspension (1 mL) was inoculated separately in artificial water samples (100 mL) (Figure 1). Then, bacteria were filtered through polycarbonate membranes (0.22 µm pore-size; Millipore, Vimodrone, Italy). Three different and consecutive experiments for each range of bacterial concentrations were carried out for both methods.

In Exposure Protocol A, 1 mL of PMA (25 µM in saline solution) was directly applied to the filter, followed by incubation in the dark for 5 min and photoactivation (500 W halogen lamp at a distance of 20 cm) on ice for 5 min. DNA was then directly extracted from the filter (with and without PMA treatment) using an Aquadien kit (Biorad, Milan, Italy) and quantified using an iQ-Check Quanti *L. pneumophila* kit (Biorad, Milan, Italy) according to the manufacturer’s instructions. The detection limit of this qPCR method was 5 genome units (GU) per well; performing DNA extract amplification in duplicate, the detection limit of the total method was 80 GU/L. The quantification limit was 15 GU/5 µL corresponding to 500 GU/L.

In Exposure Protocol B, the filters were placed in 10 mL of sterile saline solution (NaCl 0.9%) and retained cells were released by sonication for 7 min. The resulting cellular suspension was divided into two fractions (the first for qPCR and the second for PMA-qPCR). Each fraction was concentrated to 1 mL using centrifugation (16,000× *g* for 5 min). One fraction was exposed to PMA (5 min in the dark and 5 min exposure to a 500 W light at a distance of 20 cm on ice). Subsequently, all fractions were concentrated (16,000× *g* for 5 min) to approximately 100 µL. The DNA extraction and quantification were performed as previously described for Exposure Protocol A.

### 2.4. Statistical Analysis

All data generated by qPCR were analyzed by the Opticon Monitor Analysis Software version 3.4 (Biorad, Milan, Italy). To quantify the effect of PMA on heat-killed cells, Δ log values were calculated (log value qPCR − log value PMA-qPCR).

The statistical analysis was conducted with the statistical package IBM SPSS Statistics 24.0 (IBM Corporation, Armork, NY, USA). Significant differences between the doses of PMA tested for cytotoxic effect and between the two exposure protocols were assessed by ANOVA, Tukey’s multiple comparison, and a *t*-test.

## 3. Results and Discussion

Preliminary tests were carried out using different concentrations of PMA to determine the PMA concentration to be used, thereby avoiding possible cytotoxic effects. A comparison of plate counts obtained after dye exposure (on filter or in liquid) versus that obtained from non-dye exposed controls is shown in Table 1.

Exposure to all the PMA concentrations tested revealed a modest cytotoxic effect. No significant differences (*p* > 0.05, ANOVA test) in cytotoxic effect were observed among the three concentrations of PMA tested with both methods. Thus, the 25 µM PMA concentration appears to be the most suitable concentration for use because it is the highest dose that showed a limited cytotoxic effect.

**Figure 2:**
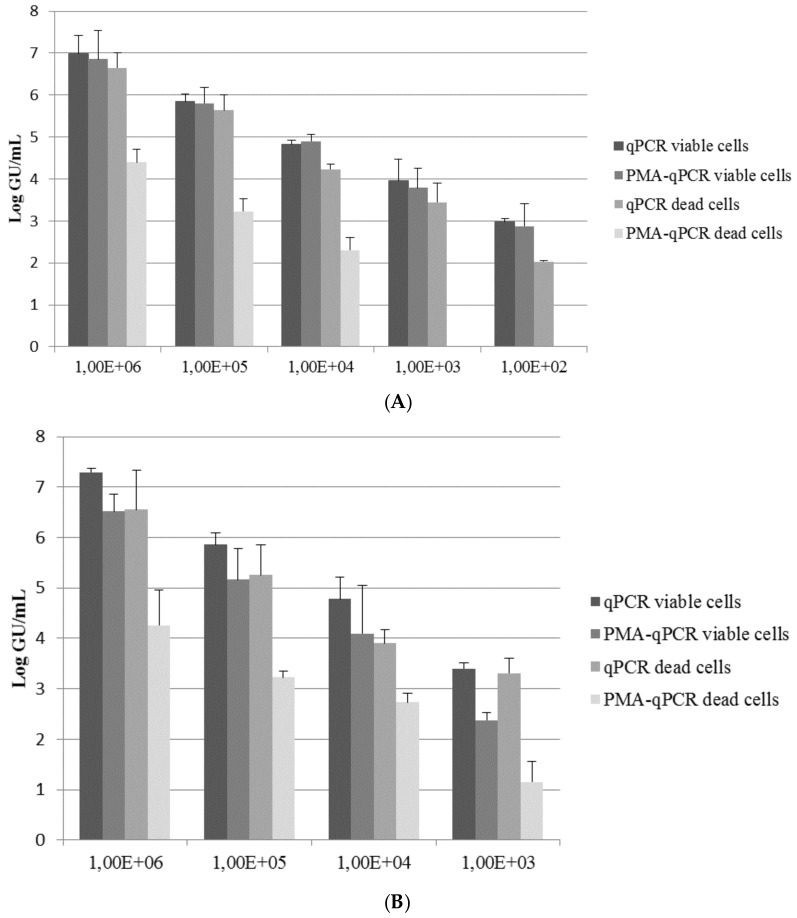
Quantification of the dilution ranges (10^2^–10^6^ colony-forming units (CFU)/mL) for viable and heat-killed *L. pneumophila* by quantitative polymerase chain reaction (qPCR) and PMA-qPCR. Each pillar represents the mean value obtained in three independent experiments for each bacterial concentration. The error bar represents standard deviations of the mean from three independent experiments. (**A**) Exposure Protocol A, on filter; (**B**) Exposure Protocol B, in liquid.

The results using Exposure Protocol A were obtained with qPCR on viable and heat-killed cells (with and without PMA) are reported in Figure 2A.

The qPCR values (viable cells) were on average 0.37 log units greater than those obtained by culture conditions (Δ log ranging from 0.44 to 0.28). This trend has been reported in other studies [6,7] and it could be related to the PCR detection method, which also includes the quantification of *Legionella* doublets or chains as individual cells, while they are counted as only 1 CFU by the culture method. Moreover, a slight reduction of genomic units with qPCR (heat-killed cells) compared to qPCR (viable cells) was observed (mean Δ log 0.52). The lower number of heat-killed cells suggests that some of the genomic DNA of the killed cells was destroyed by the heat treatment. This has also been observed by other authors, who have reported a decrease of about 0.63 log units [13]. Otherwise, low differences between qPCR and PMA-qPCR on viable cells have been revealed.

In a comparison of the data obtained from qPCR and PMA-qPCR on heat-killed *L. pneumophila* cells, PMA treatment led to an average reduction of ~2 log units for the highest inoculum concentrations (from 10^6^ to 10^4^ CFU). A similar mean reduction was reported in the studies of Ditommaso et al. [7,14,15], but they observed a dissimilarity in the ability of PMA (50 µM) to suppress the PCR signal in samples with different amounts of bacteria: increasing cell amounts resulted in higher reduction values [14]. In our study, a total reduction (3.4 log units) was reached when an initial inoculum concentration of ≤10^3^ CFU was used. A similar reduction was observed by Slimani et al. [16] in all inoculum concentrations tested, although those authors used a lower PMA concentration (6.25 µM). On the contrary, in other studies, the PMA treatment (50 µM) did not completely reduce the signal for heat-killed *Legionella*, even at a low initial inoculum concentration [14].

The results of Exposure Protocol B obtained with qPCR on viable and heat-killed cells (with and without PMA) are shown in Figure 2B.

As observed in Protocol A, the values determined by qPCR (viable cells) were on average 0.57 log units higher than those obtained by culture (Δ log ranging from 1.03 to 0.10). Moreover, a slight reduction of genomic units with qPCR (heat-killed cells) compared to qPCR (viable cells) was observed (mean Δ log 0.58). Unlike Protocol A, a slight reduction of genomic units with PMA qPCR (viable cells) compared to qPCR (viable cells) was observed (mean Δ log 0.80). PMA induces a signal reduction, which suggests the presence of a certain proportion of membrane-compromised cells within these “live” cell suspensions as reported by other authors [6]. Moreover, no amplification was obtained when the concentration of viable or dead cells was less than 10^3^ CFU. These phenomena could be related to the different steps that are necessary for recovering cells from the washing liquid, which can result in a loss of the qPCR signal.

In a comparison of the results obtained from qPCR and PMA qPCR on heat-killed *L. pneumophila*, PMA treatment resulted in a reduction that ranged between 2.29 and 1.17 log units. A similar mean reduction was reported by Chang et al. [13] when exposing *Legionella* cells (7 log) to a higher PMA concentration (50 µM) in liquid. However, in our study, no overall reduction was obtained using PMA on heat-killed cells. These results have also been reported by other authors that targeted relatively short amplicon sizes [15,17,18] such as those used in this work.

To determine which exposure method allows a higher DNA signal reduction of dead cells, reduction values obtained with Exposure Protocols A and B were compared (Table 2).

The results obtained highlighted a higher PMA-induced qPCR signal reduction for Exposure Protocol A with respect to Exposure Protocol B at all concentrations tested. A significant difference (*p* < 0.05, *t*-test) in the reduction between Protocols A and B was observed for the inoculum concentration of 1 × 10^4^ and 1 × 10^3^ CFU/mL. Moreover, an overall reduction was obtained only for Protocol A at a bacterial concentration of 1 × 10^2^ CFU/mL; on the contrary, no amplification was observed at the same concentration using Protocol B.

These results confirmed a dissimilarity in the ability of PMA to suppress the PCR signal of dead cells using different exposure protocols, highlighting a higher capability of Exposure Protocol A (filter) to differentiate live from dead cells, mainly at low concentrations.

It is important to highlight that this study was performed by inoculating artificial water samples with different concentrations of *L. pneumophila*. Since the PMA-qPCR method may overestimate the number of viable *L. pneumophila* in samples with a high load of dead microorganisms [16,19], it may be possible to evaluate the effect of non-legionellae microflora co-existing with legionellae on quantification of viable legionellae by using both methods. In order to do so, artificial samples could be spiked with other viable and dead bacteria such as *Pseudomonas* as well. Moreover, it could be interesting to compare Protocol A (on filter) with Protocol B (in liquid) in the same real water samples to confirm the results obtained in artificial samples.

## 4. Conclusions

Environmental monitoring represents an important tool for the assessment of *Legionella* contamination, and the availability of more reliable *Legionella* detection methods could be of great value for the rapid identification of contaminated water systems [11]. The capacity for live/dead differentiation may lead to a better estimate of sanitary risk and is therefore an advantage for implementing DNA-based diagnostics in routine applications.

In this study, the results obtained with both exposure protocols showed that, at high concentrations of inoculum, no overall reduction was obtained using PMA on heat-killed cells leading to an overestimation of viable *Legionella* in spiked samples. However, a comparison between the results obtained with PMA Exposure Protocol A (filter) versus B (liquid) reveals that filter exposure allows a higher PMA-qPCR signal reduction to be reached, significantly at low concentrations. This confirms the potential use of this method to quantify *L. pneumophila* in water with low contamination. The choice of this method also has the advantage of reducing the number of detection steps, thereby avoiding bacterial loss but increasing sensitivity.

## Figures and Tables

**Table 1:** Cytotoxic effect of different concentrations of propidium monoazide (PMA) on live *Legionella pneumophila* cells.

	7 µM		12.5 µM		25 µM	
	−PMA	+PMA	−PMA	+PMA	−PMA	+PMA
**Protocol A (Filter)**	6.04 ± 0.15	5.89 ± 0.14 ^a^	5.97 ± 0.10	5.84 ± 0.18 ^a^	5.25 ± 0.11	5.14 ± 0.12 ^a^
***Legionella* Reduction** *	−0.15		−0.13		−0.11	
**Protocol B (Liquid)**	6.41 ± 0.22	6.13 ± 0.20 ^a^	6.56 ± 0.18	6.23 ± 0.21 ^a^	6.66 ± 0.13	6.40 ± 0.38 ^a^
***Legionella*** **Reduction** *	−0.28		−0.33		−0.26	

^a^ The results are expressed as mean value (log colony-forming units (CFU)/mL) and standard deviation of three independent experiments for each dose; * Reduction is expressed as the relative difference between the mean log CFU/mL of the cells treated with PMA and the mean log CFU/mL of non-dye exposed controls; −PMA: non-dye exposed controls; +PMA: cells treated with PMA.

**Table 2:** PMA-induced quantitative polymerase chain reaction (qPCR) reduction of heat-killed *L. pneumophila* cells at different inoculum concentrations with Exposure Protocols A and B.

Inoculum Concentration	PMA-Induced qPCR Signal Reduction ^a^	
	Exposure Method A (Filter)	Exposure Method B (Liquid)
1 × 10^6^	2.25	2.29
1 × 10^5^	2.42	2.04
1 × 10^4^	1.93 *	1.17 *
1 × 10^3^	3.45 *	2.16 *
1 × 10^2^	2.03	nv
Mean (SD)	2.42 (0.61)	1.91 (0.51)

Three independent experiments were carried out for each bacterial concentration; nv: not valuable; * *p* < 0.05, PMA-induced qPCR signal reduction (Protocol A) vs. PMA-induced qPCR signal reduction (Protocol B), according to *t*-test; ^a^ calculated as qPCR (genome units (GU) mean of three experiments) − PMA qPCR (GU mean of three experiments) of heat-killed cells; SD: standard deviation.
